# Medical Malpractice in Wuhan, China: A 10-Year Autopsy-Based Single-Center Study

**DOI:** 10.1097/MD.0000000000002026

**Published:** 2015-11-13

**Authors:** Fanggang He, Liliang Li, Jennifer Bynum, Xiangzhi Meng, Ping Yan, Ling Li, Liang Liu

**Affiliations:** From the Department of Forensic Medicine (FH, XM, PY, LL), Wuhan University School of Medicine, Wuhan; Department of Forensic Medicine (LL), School of Basic Medical Sciences, Fudan University, Shanghai, PR China; Division of Forensic Pathology (FH, LL, LL), University of Maryland School of Medicine; Department of Pathology (JB), Johns Hopkins University Hospital, Baltimore, Maryland; and Department of Forensic Medicine (LL), TongjiMedical College, Huazhong University of Science and Technology, Wuhan, PR China.

## Abstract

Medical disputes in China are historically poorly documented. In particular, autopsy-based evaluation and its impact on medical malpractice claims remain largely unstudied. This study aims to document autopsy findings and medical malpractice in one of the largest cities of China, Wuhan, located in Hubei Province. A total of 519 autopsies were performed by the Department of Forensic Medicine, Wuhan University School of Medicine, Wuhan, China, over a 10-year period between 2004 and 2013. Of these cases, 190 (36.6%) were associated with medical malpractice claims. Joint evaluation by forensic pathologists and clinicians confirmed that 97 (51.1%) of the 190 claims were approved medical malpractice cases. The percentage of approved malpractice cases increased with patient age and varied according to medical setting, physician specialty, and organ system. The clinico-pathological diagnostic discrepancy was significantly different among various physician specialties (*P* = 0.031) and organ systems (*P* = 0.000). Of those cases involved in malpractice claims, aortic dissection, coronary heart disease, and acute respiratory infection were most common. Association between incorrect diagnosis and malpractice was significant (*P* = 0.001). This is the first report on China's medical malpractice and findings at autopsy which reflects the current state of health care services in one of the biggest cities in China.

## INTRODUCTION

Medical malpractice can present an unwelcome emotional and economic burden to the involved practitioners, patients, and patients’ families, and it is a huge problem around the world.^1–4^ According to a nationwide client base in the United States, 7.4% of all physicians annually had a malpractice claim, with 1.6% having a claim leading to a payment.^1^ The mean indemnity payment for malpractice in the United States was $274,887, and the median was $111,749.^1^ In 2010, the financial burden of medical malpractice was over $55 billion in the United States^2^. Medical malpractice is also a burden in other western countries. One insurance company from Germany reported that approximately 4500 out of 108,000 insured doctors were confronted with complaints each year, with settlement of cases in 30%, and 10% going to a civil court.^3^ Autopsy-based forensic evaluations were central and critical to the final outcome of these cases.^4–7^

Medical disputes continue unabated both in China and around the world^8–13^. In the United States, the Institute of Medicine reported that up to 98,000 patients die of preventable medical errors in hospitals each year.^14^ Unfortunately, it is prohibitively difficult to obtain such data in China. According to the rates in the United States, it was, however, optimistically estimated that at least 420,000 patients (1.3 billion people in China vs 0.3 billion in the United States) may die each year in China from preventable medical errors. In China, the number of medical complaints was rising dramatically over years; however, medical disputes remain poorly documented from a clinical perspective. In fact, medical malpractice data in China were reported only in a legal perspective,^15^ and were not accessible to clinicians; the autopsy-based evaluation and its relationship to medical malpractice claims have been rarely reported in China.

Owing to the health care system differences among China and other countries, China has witnessed region-specific features of medical malpractice. In China, clinical pathologists seldom evaluate malpractice claims (patients’ family desire evaluation by an independent party with medical knowledge, and without relationship to a hospital). Forensic pathologists work for independent institutes, which are not affiliated with any hospital. Hence, forensic pathologists seldom attend formal clinical pathology conferences (CPCs) in hospitals; however, the presence of clinical physicians may be requested at autopsy in order to state their opinions in the course of forensic evaluations. Forensic pathologists also obtain clinical consultation (from senior clinical pathologists and physicians) to ensure scientific objectivity. In addition, medical malpractice claims are usually addressed either by the Medical Association affiliated with the local Department of Health or by other authorities upon application appraisal. Forensic autopsies are always ordered by the authorities investigating a case, and a complete evaluation of the alleging case is performed by the forensic pathologists and other clinical professionals. In this setting, autopsy and the complete case evaluation are both conducted in forensic medical institutes, which avoids prejudice against involved parties. To most families, the neutrality and objectivity offered by forensic medical institutes are preferable above the Medical Associations for malpractice evaluation, as families are more likely to suspect that Medical Associations may hold similar interests with the involved hospitals. Unfortunately, no uniform registry system is currently available among forensic medical institutes in China, and the low rate of consent to autopsy by victims’ families may complicate the forensic autopsy-based evaluation. These barriers collectively contribute to the difficulty obtaining nationwide data regarding medical malpractice in China.

The Wuhan University School of Medicine, Department of Forensic Medicine, is one of the 2 most reliable and highly certified forensic medical institutes in Wuhan, Hubei Province, which is one of the biggest cities in China. It performs a substantial number of forensic evaluations of medical malpractice cases, including cases which are referred from Hubei Province and other provinces in China. In the present study, we retrospectively reviewed medical malpractice claims that were referred to this department over a 10-year period. Only closed cases that were autopsied within the above department were included. The aims of the present study were 3-fold: first, to examine the distribution of medical malpractice claims and approved cases from a clinical perspective; second, to classify types of diagnostic and medical errors, by which associations were analyzed between clinico-pathological diagnostic discrepancies and malpractice; and lastly, to identify common medical errors immediately related to untimely death. This study represents data from a single center, which maps the current status of medical malpractice in one of the biggest cities in China.

## METHODS

### Ethical Statements

The research protocols, including design and implementation of the study, were approved by the Ethics Review Board from Wuhan University School of Medicine. Informed consent was distributed to and obtained from each claimed case. Claimed cases that showed their reluctance in participating in our study by their families were excluded. Data were protected to maintain patient privacy. Each forensic evaluation was conducted by specialists without conflict of interest related to the case.

### Settings

The Wuhan University School of Medicine, Department of Forensic Medicine, is certified to practice forensic evaluation by the Department of Justice, Hubei Province, China. This department predominantly serves the area of Hubei Province and other provinces around Hubei. All protocols in the practice of forensic evaluation are in accordance with official regulations. For each case, after autopsy, a complete evaluation was conducted by a review board, which included the attending forensic pathologists and other clinical professionals.

### Study Design and Participants

In this retrospective study, a total of 519 autopsy cases were referred to and subsequently completed by the Wuhan University School of Medicine, Department of Forensic Medicine, over a 10-year period from January 1, 2004 to December 31, 2013. Among the 519 cases, 190 cases (36.6%) involved medical malpractice claims. All 190 cases routinely underwent complete autopsies, including assessment of each organ, which were formalin-fixed, paraffin-embedded, and then histopathologically examined. To guarantee the quality of autopsy assessment, only cases that met one of the following requirements were included: fresh cases that died within 24 hours; cases that were immediately kept in a freezer for no >7days after death. For this study, information including demographics, medical records, autopsy reports, investigation reports, and laboratory tests were reviewed. Cases missing any of these components were excluded. The decision for each claim was defined as approved malpractice, nonmalpractice, or undetermined. Reasons for cases being undetermined included questioned authenticity of medical documents, and termination of forensic evaluation due to settlement of a case through mediation. The decision for each case was made in consensus by the review board.

### Classification of Involved Health Care Facilities

In urban areas of China, a 3-tiered system is used to classify public hospitals, which includes community health care centers (primary), district hospitals (secondary), and city hospitals (tertiary). Similarly, there is a 3-tiered health care structure in rural areas, composed of village clinics, township health care centers, and county hospitals.^16^ However, some tertiary hospitals affiliated with well-known universities (university hospitals) have more resources than nonaffiliated tertiary hospitals and may be considered yet another tier. In addition, the widespread use of private or family clinics under the market-opening policy has even complicated the current structure of health care system.^17^ To succinctly and better classify China's current system of health care facilities, we introduced the following 4-tiered system, based on China's current situation.

Type A: Hospitals or medical centers affiliated with well-known universities, with significant teaching and research as a part of their core mission and with the most complicated cases.

Type B: Larger hospitals or medical centers with >100 beds and moderate capabilities for higher-level care, located in counties or cities.

Type C: Small hospitals with <100 beds and minimal capacity for higher level care, such as township hospitals or community health centers.

Type D: Small family or private clinics with no inpatient beds that can only perform simple clinical care.

### Classification of the Clinico-Pathological Diagnostic Discrepancies

For comparison between clinical diagnoses and autopsy diagnoses within each case, 5 principle diagnoses were extracted from clinical records and autopsy reports, respectively. These 5 diagnoses were grouped as major diagnoses (primary causes of death and the principle underlying contributors) and minor diagnoses (antecedent disorders, related diagnoses, contributing causes, or other important conditions) as has been previously described.^18–20^ Based on major and minor diagnoses, 3 forensic pathologists were assigned to independently identify the discrepancies between clinical and autopsy diagnoses of each case. A single class of discrepancy was assigned to each case. Discrepancies were classified by agreement of 3 pathologists. In a few cases for which there was no agreement, a senior pathologist uninvolved in the study was consulted. The discrepancies were assessed a second time, and reclassified if necessary. Discrepancies between clinical and autopsy diagnoses were accordingly classified by the following criteria:

Correct diagnoses: Major clinical diagnoses correctly matched major diagnoses at autopsy, despite discrepancies in minor diagnoses not directly related to cause of death or without adverse prognostic implications.

Incorrect diagnoses: Unknown major clinical diagnoses which were disclosed at autopsy, although some clinically presumed minor diagnoses were found at autopsy, or clinically presumed major diagnoses not found at autopsy, despite minor clinical diagnoses that might match autopsy minor diagnoses.

Indeterminate: Cause of death was clinically uncertain, or cause was suspected but could not be clearly diagnosed and confirmed.

### Physician Specialty

Physician specialties were classified as surgery, internal medicine, pediatrics, obstetrics, emergency medicine, and other departments, such as ophthalmology, ears, nose, and throat (ENT), dermatology, and general practice. Physicians from type D clinics are not specialized, and perform general practice.

### Classification of Medical Errors

Medical errors were classified into 5 groups as previously described by Madea and Preuss^3^:

1. Group 1: Negligence, such as omissions of necessary treatments or therapeutic omissions, delayed admission to hospital, and insufficient diagnostics.
2. Group2: Preventable complications at and/or after surgery, perioperative complications.
3. Group 3: Wrong treatment, inappropriate treatment.
4. Group 4: Mistakes in care, suboptimal care.
5. Group 5: Adverse drug events, medication errors, such as wrong drug or dose, wrong application, disregarding drug allergy, and illegible medication.

### Statistical Analysis

Cases were calculated as numbers in total with corresponding percentages when necessary. The Pearson χ^2^ and Fisher exact tests were used to assess statistically significant differences. Test results yielding 2-tailed values of *P* < 0.05 were considered statistically significant. All analysis was performed by using SPSS 16.0 software (SPSS Inc, Chicago, IL).

## RESULTS

### Distribution of Medical Malpractice Cases

**FIGURE 1:**
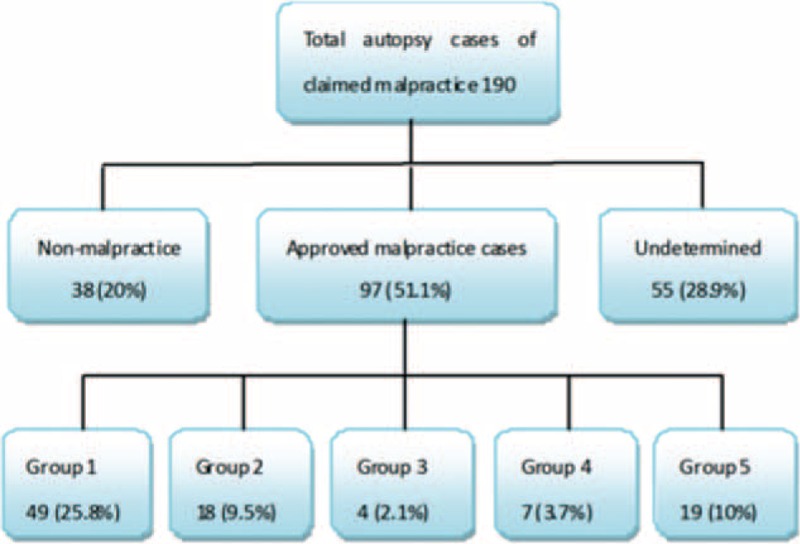
Flow chart showing how cases were selected and classified.

A total of 190 medical malpractice claims were evaluated. Of the 190 cases, 135 (71.1%) were classified as malpractice (n = 97, 51.1%) or nonmalpractice (n = 38, 20%). The remaining 55 cases (28.9%) were undetermined (Fig. 1).

**FIGURE 2:**
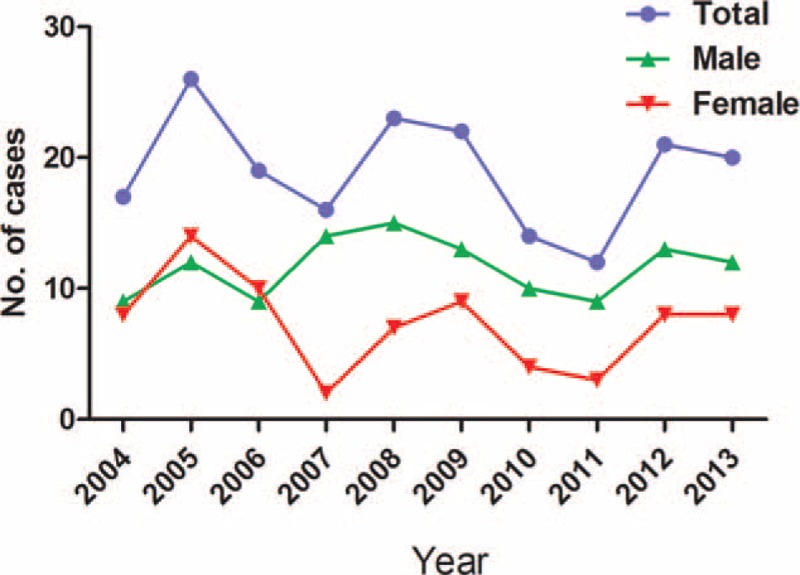
Distribution of all malpractice claims by year and sex.

The age at death ranged from newborn (1 day old) to 85 years with the mean age 31.7 ± 21.9 years and median age 35 years. The number of malpractice claims fluctuated, but generally ranged from 12 to 26 cases per year. Male patients were more common with a male: female ratio as 1.6:1 (Fig. 2).

**FIGURE 3:**
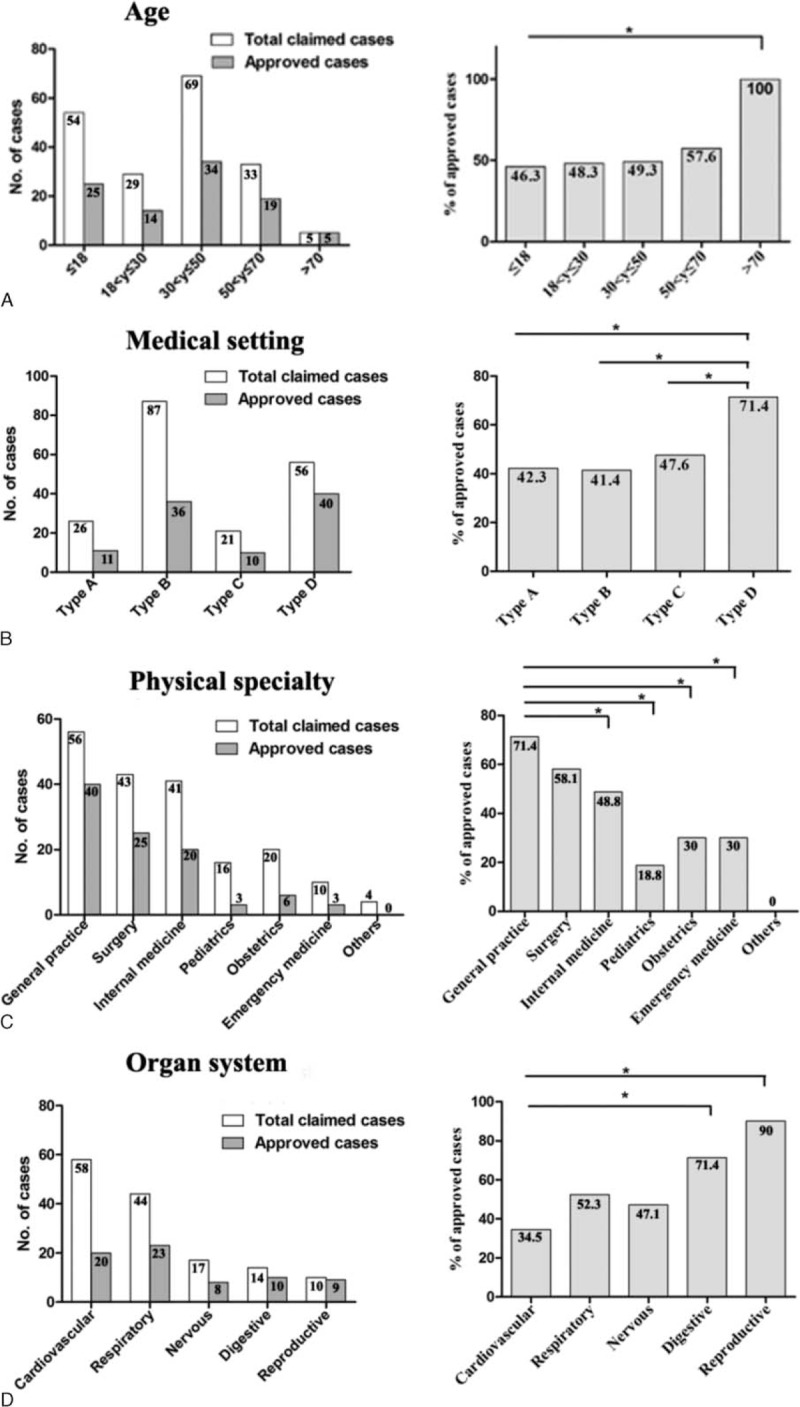
Distribution of malpractice cases by age, medical setting, physician specialty, and organ system. Number of cases and the percentage of approved malpractice were shown in the left and right panels, respectively, in each category. The percentages of approved malpractice were compared among groups (right panels). In general, the percentage of approved malpractice in elderly patients (>70 years) was significantly higher than that of other age groups. Type D hospitals were at a significantly higher risk of malpractice. General practice was a significantly riskier specialty than other specialties. The percentages of approved malpractice were remarkable in digestive and reproductive systems, which were significantly higher than that in cardiovascular system.

Of the 190 claims, patients <18 years and those between 30 and 50 years were the 2 most common age groups, which accounted for 54 (28.4%) and 69 (36.3%) cases, respectively. However, the percentage of approved malpractice increased from younger to older age groups. Of claims 46.3% were approved as malpractice in patients <18 years, whereas in the 70+ patients, all 5 claims were approved as malpractice (Fig. 3A).

Upper-tier (types A and B) hospitals were involved in more malpractice claims (n = 113, 59.5%), with type B having the most claims (n = 87, 45.8%). However, lower-tier (types C and D) hospitals were involved in a higher percentage of approved malpractice, with type D having the highest percentage (71.4%) (Fig. 3B).

Among physician specialties, general practice faced the highest number of malpractice claims (n = 56) and also had the highest percentage of approved cases (71.4%), followed by surgery (43 claims, 58.1% approved) and internal medicine (41 claims, 48.8% approved) (Fig. 3C). Among organ systems, the cardiovascular system was involved in the highest number of malpractice claims (n = 58, 30.5%); however, only 34.5% (n = 20) of these cases were approved as malpractice. The top 2 systems involved in the highest percentages of approved malpractice were the reproductive system and digestive system, 90% and 71.4%, respectively (Fig. 3D).

### Discrepancies Between Clinical Diagnosis and Autopsy Diagnosis According to Medical Setting, Physician Specialty, and Organ System

Of the 190 malpractice claims, 94 were correctly diagnosed clinically and confirmed at autopsy, 68 were incorrectly diagnosed, and 28 were indeterminate (Table 1). No significant difference in clinico-pathological diagnostic discrepancies was observed among medical settings (*P* = 0.722). Instead, significant difference was observed among physician specialties (*P* = 0.031) and organ systems (*P* < 0.001). Notably, the department of obstetrics had significantly higher correct diagnoses versus incorrect ones (*P* < 0.05). Cardiovascular diseases were more often incorrectly diagnosed than correctly diagnosed (*P* < 0.05).

**TABLE 1:**
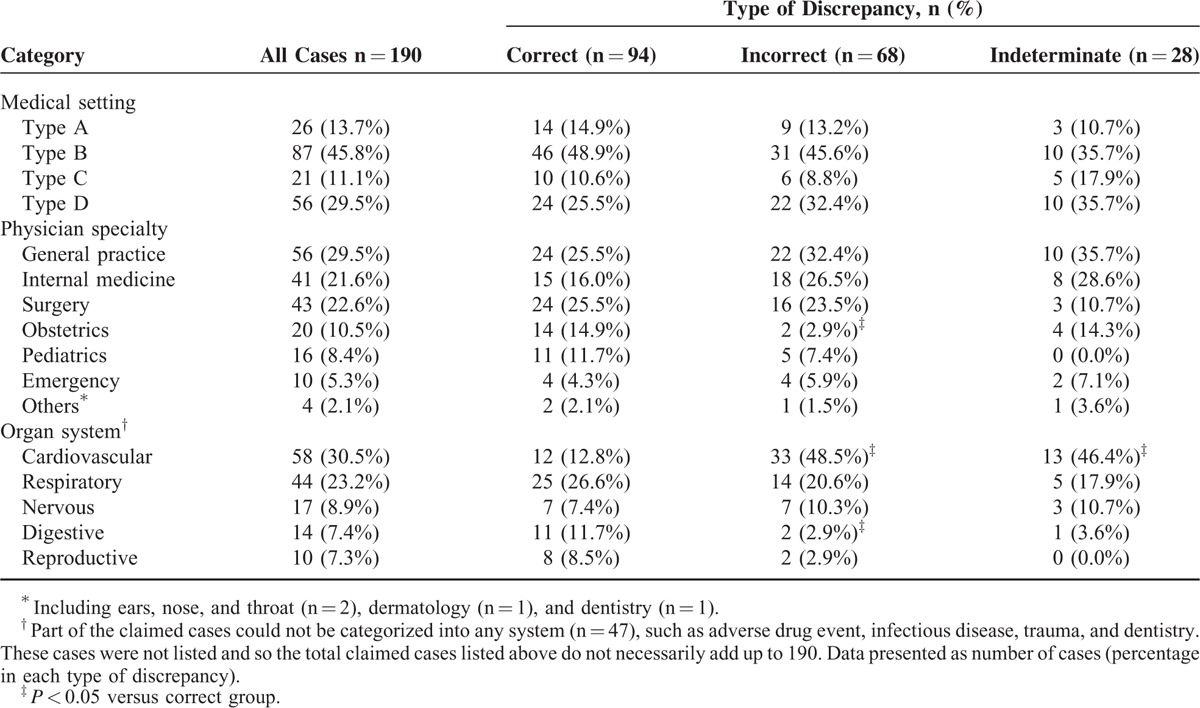
Discrepancies Between Clinical and Autopsy Diagnoses According to Medical Setting, Physician Specialty, and Organ System

### Association Between Diagnostic Discrepancy and Approved Malpractice

Among the 190 malpractice claims, diagnostic discrepancy was closely correlated with malpractice (*P* = 0.001). When a clinical diagnosis was correct, it did not always negate malpractice. That is, correct diagnoses do not necessarily create a lower risk of malpractice, as demonstrated by cases due to adverse drug events. In the 20 cases of death secondary to adverse drug events, 19 were caused by medical errors, although 15 cases were correctly diagnosed. When a clinical diagnosis was incorrect, malpractice was, however, more commonly approved (37.1% vs 15.8%, *P* < 0.05). Likewise, when the diagnostic discrepancy was indeterminate, malpractice was more often negated (31.6% vs 9.3%, *P* < 0.05) (Table 2).

**TABLE 2:**
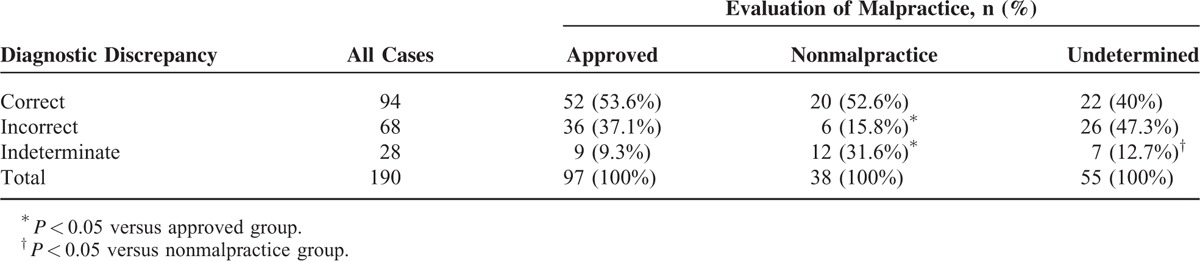
Association Between Diagnoses and Malpractice

### Types of Medical Errors Underlying the Approved Malpractice Cases According to Medical Setting, Physician Specialty, and Organ System

Of the 97 approved malpractice cases, medical errors included negligence (group 1), surgical complications (group 2), wrong treatment (group 3), mistake in care (group 4), and adverse drug event (group 5). Among these errors, groups 1, 2, and 5 were most common, accounting for 49 (50.5%), 18 (18.6%), and 19 (19.6%) cases, respectively (Table 3). With respect to medical settings, type A hospital errors were most commonly group 2, type B most commonly group 1, and type D most commonly groups 1 and5. The rate of adverse drug events was strikingly high, up to 32.5% in type D (13 of 40). General practice, which is mainly performed in Type D clinics, predominantly made errors in groups 1 and 5. The department of surgery predominantly made errors in group 2 (surgical complications). Errors from cardiovascular and respiratory systems were predominantly group 1.

**TABLE 3:**
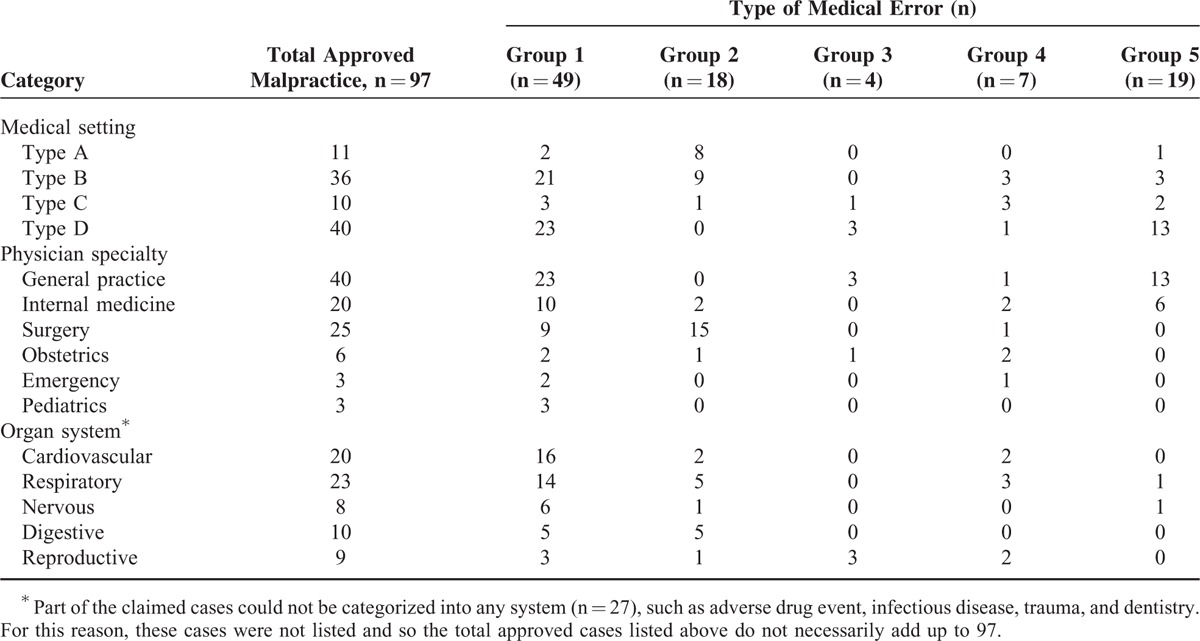
Medical Errors Associated With Approved Malpractice Cases According to Medical Setting, Physician Specialty, and Organ System

### Primary Diseases Involved in Medical Malpractice Claims

Of the 190 cases, acute respiratory infections, coronary heart disease, and aortic dissection were the most common diseases involved in claims, accounting for25 (13.2%), 23 (12.1%), and 17 (8.9%)claims respectively, and together accounted for 34.2% of all claims (Table 4). Statistics showed that diagnostic discrepancy was significantly different among the 3 diseases (*P* < 0.001). Rate of correct diagnosis was significantly high in acute respiratory infection (64%), whereas rate of incorrect diagnosis was remarkably high in aortic dissection (82.4%) and coronary heart disease (52.2%). No significant difference was observed among the 3 diseases with regard to medical setting for claimed cases (*P* = 0.230), rate of approved malpractice (*P* = 0.474), and medical settings for approved cases (*P* = 0.136).

**TABLE 4:**
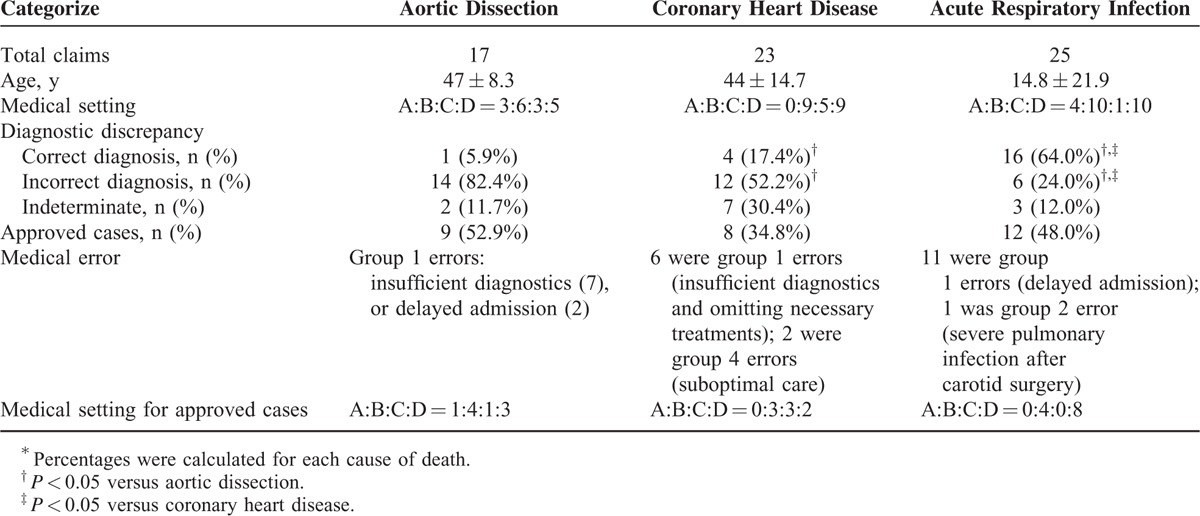
Characteristics of Malpractice Cases Due to Aortic Dissection, Coronary Heart Disease, and Acute Respiratory Infection^∗^

Of the 17 claims due to aortic dissection, the mean age was 47 ± 8.3 years, representing a midlife incidence. Nine (52.9%) cases were approved malpractice due to group 1 error (7 insufficient diagnostics, 2 delayed admission), making it the highest percentage of approved cases among the 3 diseases. Furthermore, only 1 (5.9%) case of aortic dissection was correctly diagnosed antemortem. The high incidence of malpractice due to aortic dissection was likely attributable to the low diagnostic rate, as supported by the finding that the 9 approved malpractice cases were all caused by group 1 errors.

Of the 23deaths due to coronary heart disease, the mean age was 44 ± 14.7 years, also representing a generally midlife incidence. Only 4 (17.4%) cases were correctly diagnosed antemortem and 8 (34.8%) cases were approved as malpractice. Among the 8 malpractice cases, 6 involved in group 1 error, and 2 involved in group 4 error (1 patient in psychotic and manic status was continuously constrained by medics with tight-bind bands in order to perform intravenous injection and found unresponsive the next day; another patient hospitalized in a drug rehabilitation center was found decomposed due to negligence).

Although the rate of correct diagnosis was fairly high (64%), of the 25 claims due to acute respiratory infection, 12 (48.0%) were approved as malpractice. Eleven out of the 12 approved cases were caused by group 1 errors and 8 were from type D clinics. Although these cases were generally correctly diagnosed, they were considered group 1 errors because of delayed admission to hospital after diagnosis. These patients kept seeing primary practitioners even when the medical intervention did not improve their conditions. Among the medical settings, type D had the highest rate of approved malpractice, with 8 out of 10 claims approved. Strikingly, among the 12 approved cases, 8 were infants under 3 years old. Of these 8 infant cases, 6 occurred associated with type D and 2 associated with type B hospitals, all involved in group 1 errors. The 2 infant deaths associated with type B hospitals were correctly diagnosed with pneumonia and were treated accordingly. However, 1 infant had an atrial septal defect which was not detected, a condition which significantly contribute to its death. The other infant was approved due to a missed diagnosis of myocarditis, which developed after hospitalization.

Of note, though only a small number of cases were associated with claims among the patients who were >70, all 5 deaths were caused by medical errors (Table 5). The involved health care facilities included types A (n = 2), B (n = 2), and D (n = 1). Three (60%) died of preventable surgical complications (group 2 error). One died of asphyxia due to aspiration of food (group 4 error). Another patient from a type D clinic died of anaphylactic shock after no prior skin test (group 5 error).

**TABLE 5:**
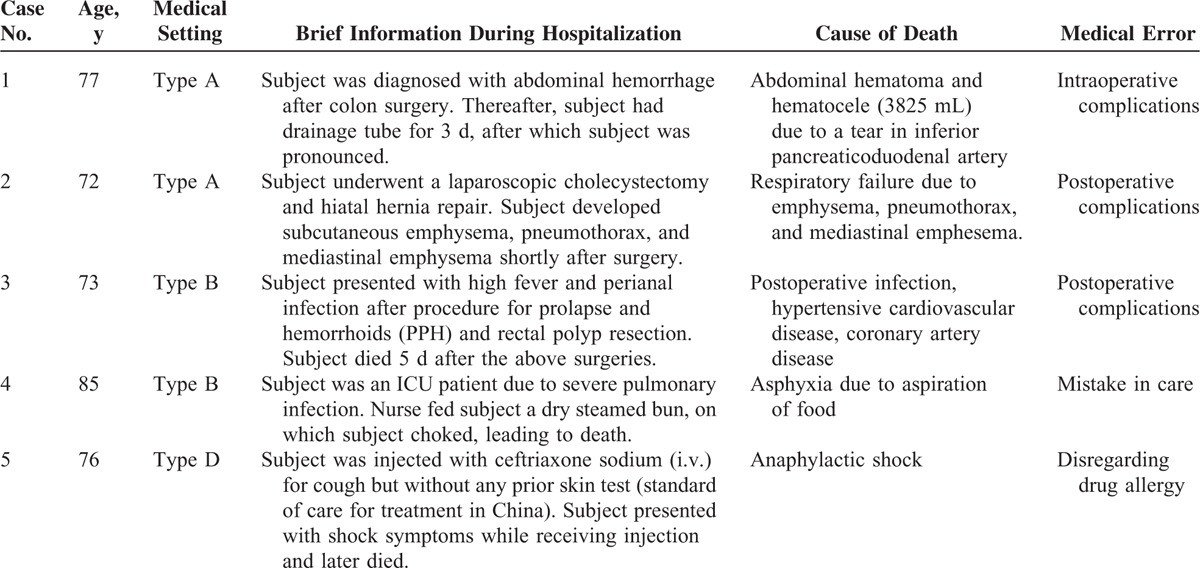
Characterization of the 5 Malpractice in Patients >70 Years Old

## DISCUSSION

### The Incidence of Malpractice

Although China has made regulatory changes in medical malpractice and greatly improved the health care system, disputes between patients and doctors have, unfortunately, increasingly intensified in the past decades.^21,22^ This is reflected by the present study. 36.7% of forensic evaluations performed in the studied institute were medical malpractice claims over the past 10 years. This is significantly higher than other countries, such as Germany, where only maximally 20% of the total autopsies were involved malpractice claims.^3^ Among the malpractice claims, the rate of approved cases was also strikingly high (51.1%). According to the annual report of the arbitration committees of the medical councils in Germany, only maximally 28.6% of malpractice claims were confirmed,^3^ whereas in the United States, there were even fewer confirmed cases.^1,12^ One possible reason for the high rate of confirmed cases is that not all unnatural deaths are mandated to have a forensic autopsy in China. Instead, substantial numbers of malpractice claims are addressed through mediation by a third party before they proceeded to authorities or to the judicial department. The referral cases to forensic medical institutes were, therefore, more likely to be complex. The high rate of approved malpractice also suggested that medical technologies in diagnostics, surgery, care, and medication still have room for improvement.

Of note, 55 claims in the present study were undetermined due to the questioned authenticity of medical documents or early termination of a forensic evaluation after the settlement of a claim by mediation. Some hospitals or individual medical personnel in China may forge or change original medical documents before they allow patients’ families to access to the medical records.^15^ This may be done to avoid the adverse effects on reputation if exposed to the public for any malpractice claims (called “malpractice crisis”), even if some claims turn out to be nonmalpractice when evaluated. The fear of any adverse effects on reputation also promotes the mediation of malpractice claims by a third party. In a third party mediation, if claimed compensation was acceptable, hospitals often agreed to settle privately. This was why many cases were terminated early while they were still under forensic evaluation.

### Characteristics of Malpractice Claims and Approved Cases

Although no increase in claims was observed year to year (Fig. 2), the percentage of approved cases increased with patients’ age, ranging from 46.3% in younger patients (≤18 years) to 100% in 70+ patients (Fig. 3A). Young patients (≤18 years) and middle-aged patients (30–50 years) had the most claims (54 and 69 cases, respectively); however, the percentage of approved claims did not parallel these findings. Instead, all of the 5 older patients were confirmed to die after medical errors, among which 60% were attributed to preventable surgical complications (Table 5). Surgery is one of the top medical-risk specialties in China and around the world.^1,11,13^ This is further demonstrated by our observation that surgical cases had the second highest rate of claims and approved cases (Fig. 3C). However, these 5 deaths were not caused by patients’ physiological status, which most often accounts for complications among elder patients,^23^ but by preventable errors, surgical complications and medical care were the main attributors.

Upper-tier hospitals were involved in more malpractice claims regardless of their higher-level medical resources and skills. Patients’ unrealistic expectations from these upper-tier (especially type B) hospitals may be one explanation for this phenomenon. With advanced health care technology, physicians in upper-tier hospitals are frequently called to answer for any result falling short of patient expectations. This is common both in China and in other countries.^11,24^ Another possible explanation may be that upper-tier hospitals are called on to perform care for patients with greater complexity due to their better resources. The complicated diseases addressed by physicians from upper-tier hospitals may lead to a higher risk of malpractice, so the percentage of approved malpractice involved in these hospitals was not significantly lower than the percentage involved in lower-tier hospitals (Fig. 3B).

Approved medical malpractice in the type D hospitals (71.4%) was strikingly remarkable in the present study. Moreover, practitioners (general practice) in type D hospitals frequently made errors in diagnosis and treatment of common diseases, such as respiratory infections, as in the 8 approved cases out of 10 claims due to acute respiratory infections (Table 4). In addition, 16 out of the 20 claims due to adverse drug events were from type D clinics, with the most approved malpractice (16 in 40 cases, 40%) due to adverse drug events (data not shown). These findings suggest the lack of strict criteria for drug administration or lack of resources to care for those who have adverse reactions to prescribed drugs. Another possible explanation was the presence of illegal or unqualified private clinics scattered in rural and urban areas.^25^ This is an issue that certainly needs to be addressed in China. In addition, physicians must be careful not to be overconfident of diagnoses and treatments without needed testing (ie, x-ray, laboratory tests), and physicians must be mindful not to make decisions based on potential financial benefits, but on what is best for patients. Infants, in particular, should be treated with great care.

### Association Between Clinico-Pathological Diagnostic Discrepancy and Approved Malpractice

A total of 94 cases were correctly diagnosed, representing only 49.5% of those involved in malpractice claims. The clinico-pathological diagnostic discrepancy rate was significantly different among various physician specialties (*P* = 0.031) and organ systems (*P* < 0.001). Interestingly, correct diagnosis is not necessarily associated with less risk of malpractice (Table 2). Although the department of obstetrics has significantly higher correct diagnoses, this did not correlate with a low risk of malpractice claims. Instead, the department of obstetrics and gynecology was one of the top 3 medical-risk specialties, as previously reported.^1^ Also demonstrating lack of association of correct diagnosis with the lack of malpractice were the 20 claims due to adverse drug events, among which 19 cases were approved malpractice despite 15 correct diagnoses.

On the contrary, incorrect diagnosis is always associated with high risk of malpractice (Table 2). Incorrect diagnosis directly resulted in wrong treatment or medication administration and consequently caused poor medical outcomes. Cardiovascular disease, for instance, was more often incorrectly diagnosed (Table 1), as with aortic dissection and coronary heart disease, with an incorrect diagnosis rate of 82.4% and 52.2%, respectively (Table 4). Among the 9 aortic dissection cases that were confirmed as malpractice, 7 cases were caused by insufficient diagnostics. In the 8 malpractice cases due to coronary heart disease, 6 were caused by diagnostic errors (Table 4). Our findings were consistent with previous reports,^5,26^ and strongly suggested that incorrect diagnosis led to a high risk of malpractice. Of note, malpractice may result from, but are not limited to, an incorrect diagnosis. The significant association between incorrect diagnosis and malpractice does not necessarily indicate causality. When an incorrect diagnosis is unfortunately made, malpractice might be avoided if other corrections are made in a timely fashion.

Interestingly, when a diagnosis is uncertain, or even suspected but not confirmed before death (diagnostic discrepancy as indeterminate), malpractice may be negated. This may be due to the fact that physicians would rather make all relevant interventions in order not to miss anything. Under this condition, all possible testing may be done to pursue a correct diagnosis and medical procedures are hence reasonable.

### Common Diseases Involved in the Malpractice Cases

Respiratory infections, acute aortic dissection, and coronary heart diseases are most commonly involved in malpractice claims.^27^ In the present study, acute respiratory infections and cardiovascular diseases were, likewise, at high risk of involvement in malpractice. Malpractice due to these 3 primary diseases mainly occurred in young or middle-aged patients (Table 4), partially accounting for the high rate of approved malpractice involving younger patients, though their risk was still lower relative to older patients (Fig. 3A). Failure/delay in diagnosis was the most common contributing risk factor in this study, as assessed by previous reports.^28^ Rate diagnostic discrepancy was significantly different among the 3 diseases. The rates of correct diagnoses were 5.9% for aortic dissection and 17.4% for coronary heart disease, which were low relative to the incorrect diagnosis. Contributors to the low rate of correct diagnosis included insufficient diagnostics and delayed admission. Variations in clinical manifestations were also significant contributors.^29^ Patients with these diseases might present with atypical and nonspecific symptoms that could be confused with other diseases. Hence, it is essential for the physician to recognize patients who are more likely to present atypically, and to aggressively pursue the diagnosis of these diseases.

Malpractice claims due to acute respiratory infections were common in infants, particularly in type D clinics. Although the rate of correct diagnosis was relatively high (64%), timely admission to hospitals for intensive observation was not accomplished, with 11 deaths caused by delayed admission to hospitals or intensive care. The common nature of respiratory infections in infants was their rapid progression if not controlled.^30^ Referral to an upper-tier hospital should be considered early in infants with rapidly progressive respiratory symptoms.

## CONCLUSIONS

Upper-tier hospitals are involved in more malpractice claims, whereas lower-tier health care centers have a higher risk of malpractice. General practice, surgery and internal medicine physicians are at high risk of malpractice. Special attention should be paid to elderly patients and patients with acute respiratory infection, aortic dissection, and coronary heart disease. Correct diagnosis is not necessarily associated with less risk of malpractice; however, incorrect diagnosis always results in a high risk of malpractice. When a diagnosis is uncertain or suspected, a wide range of diagnostic considerations should be investigated and possible interventions should be made before discharging the patients. Medical negligence, preventable surgical complications, and adverse drug events/mediation errors are the most common events involved in malpractice. There is much room for improvement in medical technologies in diagnostics, surgery, care, and medication, as malpractice continues to be a problem in China.

### Limitations

The current study represented a single-center, retrospective review of malpractice cases over a 10-year period. There was an inherent selection bias as unmediated, complex cases tended to be referred for a forensic autopsy and systemic judicial evaluation. The current classification system of clinico-pathological diagnostic discrepancy does not fully convey the severity of diagnostic discrepancies; however, we attempted in this study to highlight how a diagnostic discrepancy is related to malpractice. A classification system with more groups (class I, II, III, IV, V, and VI) had previously been described^19^; however, only 190 claimed cases were included in this study, if we used the classification system based on severity, more cells in Tables 1 and 2 would have values <5, not to speak of more cells with values as zero. Hence, the statistical errors would be magnified for Tables 1 and 2 if more categories were made. To address this dilemma, future multicenter studies in China with large sample sizes are needed to convey the severity of diagnostic discrepancies and medical malpractice in China.
